# Aseptic Meningitis as an Immune-Related Adverse Event after Pembrolizumab

**DOI:** 10.1155/2019/7183747

**Published:** 2019-11-04

**Authors:** Gian Lima, Adriana Kahn, Shashank Sama, Jacqueline Savage

**Affiliations:** Department of Internal Medicine, University of Connecticut, Hartford, CT, USA

## Abstract

**Introduction:**

The management of patients with advanced malignancies is challenging, although recent advances with immunotherapy have shown better outcomes. Pembrolizumab has been associated with a variety of immune-related side effects, but the occurrence of aseptic meningitis is rare.

**Case:**

A 55-year-old male with a history of metastatic lung adenocarcinoma previously treated with pembrolizumab presented with persistent severe headaches and photophobia. Subsequent workup with cerebrospinal fluid analysis showed elevated opening pressure, increased nucleated cells with 30% lymphocytes, elevated protein levels, and normal glucose levels. The patient was started on high doses of IV steroids and progressed with significant improvement of his symptoms.

**Discussion:**

Given the rarity of this side effect, this case is a reminder that immune checkpoint inhibitors can cause aseptic meningitis and its early recognition is important for initiation of therapy with steroids and prompt discontinuation of the immunotherapy agent.

## 1. Introduction

Immune checkpoint inhibitors have improved outcomes for patients with advanced malignancies; however, their use is limited by immune-related adverse events (irAEs). Of these, neurological irAEs are infrequent, with rare instances of Guillain-Barre syndrome, myasthenia gravis, posterior reversible encephalopathy syndrome, enteric neuropathy, transverse myelitis, pancerebellitis, autoimmune encephalitis, and aseptic meningitis previously reported [1]. Aseptic meningitis as an irAE is exceedingly rare, with only two prior reports described in the literature. We describe a case of aseptic meningitis in a patient with metastatic non-small cell carcinoma of the lung while on treatment with an anti-programmed cell death protein 1 (anti-PD1) inhibitor, pembrolizumab.

## 2. Case Report

A 55-year-old gentleman presented with acute, bilateral, throbbing frontal headache, with an intensity 9/10, starting 3 days prior to admission and progressively worsening despite acetaminophen. It was associated with photophobia, but not with fever, chills, nausea, vomiting, syncope, seizures, or focal neurological symptoms. His past medical history was significant for metastatic lung adenocarcinoma, with a resected posterior left parietal lobe brain metastasis a year prior, followed by stereotactic radiosurgery and 11 cycles of intravenous (IV) pembrolizumab, 200 mg every 3 weeks, resulting in partial response to the immunotherapy. His last dose was 3 weeks prior to admission, when pembrolizumab was stopped when he developed grade 4 autoimmune hepatitis (with alanine aminotransferase (ALT) and aspartate aminotransferase (AST) greater than 1000 IU/L) and he was treated with a course of oral steroids.

On presentation, his vital signs and systemic physical exam were unremarkable. Neurologic examination revealed intact cranial nerves and motor and sensory exams. He did not have neck stiffness or signs of meningeal irritation. Head computed tomography scan without contrast ruled out acute intracranial pathology. Cerebrospinal fluid (CSF) analysis demonstrated elevated opening pressure of 22 mmHg, 11 nucleated cells (30% lymphocytes and 58% monocytes), elevated protein concentration of 75 mg/dL, normal glucose levels, and negative cytology for neoplastic cells. He was started on antibiotics, which were subsequently discontinued after infectious workup, including blood and CSF cultures, returned negative. CSF viral panel, fungal culture, and India ink preparation were also negative. Brain magnetic resonance imaging with and without gadolinium revealed nonspecific enhancement in the region of tumor resection, without leptomeningeal enhancement or evidence of tumor recurrence.

The patient's headache persisted despite opioids but significantly improved after IV dexamethasone (initial dose of 10 mg then 6 mg every 6 hours), and one day after initiation of steroids, the headache completely resolved. Given the negative infectious workup and responsiveness to steroids, as well as recent irAE (hepatitis), he was diagnosed with grade 3 aseptic meningitis (due to severe symptoms but non-life-threatening presentation) as an irAE from pembrolizumab. He was discharged on oral dexamethasone 6 mg every 6 hours to complete a taper course as outpatient. On 6-month follow-up, the patient remained stable clinically and staging scans showed no more evidence of active disease. Due to the multisystemic adverse events experienced by this patient (initially hepatitis followed by aseptic meningitis), associated with continuous response to therapy achieving complete response despite being off pembrolizumab, decision was made to discontinue pembrolizumab.

## 3. Discussion

IrAEs can be seen across multiple organ systems with varying presentations [1]. Analysis of 9,208 patients from 59 trials reported 3.8% overall incidence of neurologic irAEs in patients receiving anti-cytotoxic T-lymphocyte-associated protein 4 (CTLA-4) antibodies, 6.1% with anti-PD-1 antibodies, and 12.0% with a combination of both [2]. However, most neurological irAEs are mild to moderate and the incidence of severe or life-threatening irAEs was around 1% [2]. Review of the current literature indicates that only two cases of aseptic meningitis have been reported with immunotherapy agents—a patient with melanoma on ipilimumab and another with triple negative breast cancer on pembrolizumab [1, 3, 4]. In our case, the etiology of the aseptic meningitis was felt to be due to pembrolizumab given the negative infectious workup, prior irAE (hepatitis), improvement of the patient's symptoms with steroids, and absence of recurrence since discontinuation of pembrolizumab.

Patients undergoing immunotherapy with abnormal neurological findings need extensive workup to rule out other etiologies such as progression of cancer, seizure disorder, bacterial or viral infection, and metabolic derangements [1]. Steroids are recommended for patients with strong suspicion of aseptic meningitis as an irAE, in addition to the immediate cessation of the immunotherapy agent [1]. Refractory cases may require escalation to pulse-dose steroids in addition to IV immunoglobulin or plasmapheresis [1]. In our case, for the steroid therapy, we chose dexamethasone as we did not have all final results of the infectious workup at that moment and considered the existing data supporting the use of dexamethasone as the first line for bacterial meningitis.

Additionally, there is now evidence that patients with response to anti-PD-1 antibodies are more likely to report irAEs irrespective of the duration of exposure to the immunotherapy agent, and systemic corticosteroid use did not appear to affect the duration of response to immunotherapy [5]. This is in line with our patient's case, who had two irAEs, was exposed to steroids, and continued to have a good response to therapy with no evidence of disease despite treatment discontinuation. In summary, aseptic meningitis is a rare but known complication of immunotherapy and clinicians should have a high index for suspicion in their oncologic patients.

## References

[1] Brahmer J. R., Lacchetti C., Schneider B. J. (2018). Management of immune-related adverse events in patients treated with immune checkpoint inhibitor therapy: American Society of Clinical Oncology clinical practice guideline. *Journal of Clinical Oncology*.

[2] Cuzzubbo S., Javeri F., Tissier M. (2017). Neurological adverse events associated with immune checkpoint inhibitors: review of the literature. *European Journal of Cancer*.

[3] Bot I., Blank C. U., Boogerd W., Brandsma D. (2013). Neurological immune-related adverse events of ipilimumab. *Practical Neurology*.

[4] Nanda R., Chow L. Q. M., Dees E. C. (2016). Pembrolizumab in patients with advanced triple-negative breast cancer: phase Ib KEYNOTE-012 study. *Journal of Clinical Oncology*.

[5] Maher V. E., Fernandes L. L., Weinstock C. (2019). Analysis of the association between adverse events and outcome in patients receiving a programmed death protein 1 or programmed death ligand 1 antibody. *Journal of Clinical Oncology*.

